# Investigating the Neuroprotective, Hepatoprotective, and Antimicrobial Effects of Mushroom Extracts

**DOI:** 10.3390/ijms26178440

**Published:** 2025-08-29

**Authors:** Menna-Allah E. Abdelkader, Hatungimana Mediatrice, Zhanxi Lin, Christopher Rensing, Mohamed M. Yacout, Dongmei Lin, Sarah A. Aggag

**Affiliations:** 1 China National Engineering Research Center of Juncao Technology, College of Life Sciences, Fujian Agriculture and Forestry University, Fuzhou 350002, China; menna.elsayed@alexu.edu.eg (M.-A.E.A.); mediatunga@gmail.com (H.M.); lzxjuncao@163.com (Z.L.); 2 Department of Genetics, Faculty of Agriculture, Alexandria University, Alexandria 21545, Egypt; mohamed.abdyacout@alexu.edu.eg; 3 Rwanda Agriculture and Animal Resources Development Board, Kigali 5016, Rwanda; 4 Institute of Environmental Microbiology, Fujian Agriculture and Forestry University, Fuzhou 350002, China; crensing94@gmail.com

**Keywords:** mushroom, D-galactose, autophagy, antibacterial, telomere length, neurodegeneration-related genes, *IL* genes

## Abstract

Mushrooms, renowned for their nutritional value and bioactive compounds, offer potential health benefits, including antioxidants and anti-aging properties. Aging, characterized by cellular and tissue decline, is often associated with autophagy dysfunction, a crucial cellular cleaning process. This study aimed to investigate the neuroprotective, hepatoprotective, and antimicrobial properties of extracts from four medicinal and edible mushrooms: *Ganoderma lucidum*, *Hericium erinaceus*, *Pleurotus ostreatus*, and *Agaricus bisporus*. The protein, total phenol, and flavonoid content of mushroom extracts were determined. Aging was induced with 120 mg/kg D-galactose and treated with 500 mg/kg mushroom extracts. The study evaluated liver enzyme levels, histopathological changes in liver and brain tissues, gene expression correlated to neurodegeneration (*SEPT5*-*SV2B*-*ATXN2*-*PARK2*), telomere length, and immunomodulatory and pro-inflammatory (*IL-2*-*IL-4*-*IL-6*) gene expression pathways. Additionally, the antimicrobial potential of mushroom extracts was assessed against several bacteria (*Lysinibacillus odyssey*, *Lysinibacillus fusiformis*, *Klebsiella oxytoca*, and *Escherichia coli*) using agar well diffusion and lowest minimum inhibitory concentration (MIC) methods. By exploring these diverse aspects, this study aimed to provide a foundation for a better understanding of the potential of mushrooms as natural neuroprotective, hepatoprotective, and antimicrobial agents and their potential applications in human health. Results indicated that all mushroom extracts effectively mitigated oxidative stress. *Agaricus bisporus* exhibited the highest protein and flavonoid content, and *Pleurotus ostreatus* displayed the highest phenolic content. Notably, *Hericium erinaceus* and *Ganoderma lucidum* extracts demonstrated significant neuroprotective and hepatoprotective properties against D-galactose-induced aging, as evidenced by histopathological examination. All extracts exhibited a significant decrease (*p* < 0.001) in liver function (serum levels of aspartate aminotransferase (GOT) and alanine aminotransferase (GPT)) and showed immunomodulatory and anti-inflammatory effects, characterized by upregulated *IL-2* and *IL-4* gene expression and downregulated *IL-6* gene expression. *Hericium erinaceus* demonstrated the most pronounced upregulation (*p* < 0.001) of *SEPT5*, *SV2B*, and telomere length gene expression, suggesting potential anti-aging effects. Furthermore, all mushroom extracts displayed antimicrobial activity against the tested bacterial strains, except *Hericium erinaceus*, which exhibited antibacterial activity solely against *E. coli*. *Agaricus bisporus* exhibited the largest inhibition zones (22 ± 0.06 mm) against *Lysinibacillus odyssey*, while *Hericium erinaceus* displayed the largest inhibition zone against *E. coli*. The MIC value was observed with *Agaricus bisporus* extract against *Lysinibacillus odyssey* (1.95 ± 0.16 mg/mL). *Lysinibacillus fusiformis* exhibited the highest resistance to the tested mushroom extracts. These findings suggest that these edible and medicinal mushrooms possess a wide range of health-promoting properties, including neuroprotective, hepatoprotective, and antimicrobial activities. Further research is needed to fully understand the underlying mechanisms and optimize applications. However, our results provide a strong foundation for exploring these mushrooms as potential natural agents that promote overall health and combat age-related decline.

## 1. Introduction

The search for natural sources of functional nutrients has grown popular due to an increased risk of fatal illnesses in humans [1]. As a result, it is critical to study mushrooms, which have long been a significant component of the human diet. These unique fungi are rich in vitamins, minerals, proteins, and bioactive compounds, including flavonoids and phenolic compounds, which have a variety of pharmacological properties [2,3,4,5]. The species investigated in this study, including *Agaricus bisporus*, *Ganoderma lucidum*, *Pleurotus ostreatus*, and *Hericium erinaceus*, are well documented to contain these essential nutrients. Given the growing demand for functional foods, edible and medicinal mushrooms are gaining recognition as valuable natural resources for promoting health and well-being [6].

Numerous scientific studies have demonstrated the diverse therapeutic potential of mushrooms. For over 2000 years, the mushroom *Ganoderma lucidum* (Reishi) has been used therapeutically in Asia. It contains bioactive compounds like triterpenoids and polysaccharides that have been shown to offer a range of health benefits [7,8]. These include neuroprotection against cognitive decline and diseases such as Alzheimer’s and Parkinson’s [9,10,11], as well as anti-cancer, anti-hypertension, and anti-atherosclerosis properties [12,13]. The mushroom also enhances cognitive function, bolsters both innate and adaptive immunity [14], and has anti-amnesic, anti-inflammatory [15], antioxidant, and antimicrobial effects [16]. Its components, such as triterpenoids and chitosan, have demonstrated strong antibacterial activity against various pathogenic bacteria, including *Staphylococcus aureus*, *Escherichia coli*, *Klebsiella pneumonia*, and *Pseudomonas aeruginosa* [16,17,18].

Lion’s Mane mushroom (*Hericium erinaceus*) shows great promise for treating neurological disorders. It produces neurotrophic molecules that are able to cross the blood–brain barrier, protecting against and delaying neuronal cell death in diseases like Alzheimer’s and Parkinson’s [19,20]. This mushroom also boosts cognitive function by promoting the creation of new neurons (neurogenesis), strengthening synaptic connections (synaptic plasticity), and reducing oxidative stress and inflammation in the brain, which enhances memory and learning [21,22]. Beyond its neurological effects, it also has antioxidant, anti-inflammatory, and hepatoprotective properties [23,24,25] and contains powerful antibacterial compounds, including phenols and fatty acids [26], that fight a wide range of bacteria [27,28,29,30].

Furthermore, interest in edible mushrooms is growing due to their culinary and medicinal properties [4]. The oyster mushroom (*Pleurotus ostreatus*) is one of the most cultivated mushrooms globally and offers a wide range of health benefits [31]. It has been shown to have anti-inflammatory, immune-boosting, anti-tumor, antimicrobial, and anti-diabetic effects [32,33,34,35]. Additionally, this multifaceted mushroom acts as a powerful antioxidant, protecting against cellular damage from free radicals and helping to prevent chronic illnesses such as diabetes, neurological diseases, and cancer [36,37]. Studies have shown that extracts from *P. ostreatus* are rich in phenols, which are associated with their antioxidant properties [38]. Oyster mushrooms also possess antimicrobial properties. Secondary metabolites like tannins and phenolic compounds in the mushroom have been shown to inhibit a wide range of pathogenic bacteria, encompassing both Gram-positive and Gram-negative bacteria [39,40,41]. Specific proteins within the mushroom, such as pleurostrin and eryngin, have also demonstrated potent antibacterial and antifungal activity [42].

The common button mushroom (*Agaricus bisporus*) is a popular edible mushroom with significant health benefits [43]. It has been shown to have anti-aging, anti-inflammatory, immune-regulating, and anti-cancer properties, and may help prevent cognitive decline and other age-related diseases [44,45,46]. Studies suggest that *A. bisporus* is able to reduce oxidative stress in the brain, which is a factor in neurodegenerative illnesses [47]. In aged mice, a polysaccharide from this mushroom improved memory and reduced both inflammation and oxidative stress in the brain [46]. Furthermore, the mushroom’s rich phytochemical composition, including tannins, flavonoids, and β-glucans, gives it broad-spectrum antimicrobial properties against bacteria such as *Staphylococcus aureus* and *Bacillus cereus* [48,49]. These combined properties make it a valuable functional food for various health applications.

Aging is a multifaceted biological process characterized by a progressive cellular and tissue function. This process can be accelerated by factors such as D-galactose, which induces a state of oxidative stress and mimics the physiological characteristics of aging in animal models [50]. The brain is particularly vulnerable to this oxidative damage, which leads to neurodegenerative processes and an increased risk of age-related neurological decline [51]. This oxidative burden has been shown to damage DNA, proteins, and lipids, weaken the immune system, and contribute to a wide range of age-related diseases [2]. The immune system itself was shown to undergo age-related changes, with chronic inflammation playing a significant role in accelerating the aging process [10]. Emerging research has highlighted the involvement of specific genes, including *Sv2b*, *Park2*, *Sept5*, *Atxn2*, *Pink1*, and *Gabbr2*, in neurological dysfunction and neurodegenerative diseases [52,53]. In this context, we selected specific genes to evaluate the effects of our mushroom extracts. Genes such as *SEPT5*, *SV2B*, *ATXN2*, and *PARK2* were chosen to assess neuronal dysfunction, while *IL-2*, *IL-4*, and *IL-6* were included to quantify the inflammatory response. Furthermore, telomere shortening is a key biomarker of cellular senescence and aging, which can be accelerated by oxidative stress [54]. As a result, researchers and healthcare professionals worldwide are focused on developing strategies to mitigate the effects of aging and promote healthy aging throughout the lifespan [55].

Compounding these aforementioned challenges, the global health landscape is threatened by antimicrobial resistance (AMR), where the effectiveness of antibiotics is waning [56]. This alarming trend, responsible for an estimated 700,000 deaths annually in 2014, is projected to claim 10 million lives by 2050 if left unchecked. As a result, it has become important to protect the integrity of the antimicrobial agents presently in use, given that the development of new antimicrobials has slowed in recent decades [57]. The urgent need for novel antimicrobial agents has spurred interest in natural alternatives like medicinal mushrooms.

Therefore, this study aims to investigate the neuroprotective, hepatoprotective, and antimicrobial effects of four specific mushroom extracts. We hypothesize that these mushroom extracts will mitigate D-galactose-induced aging by modulating neurodegeneration and inflammatory gene expression, preserving telomere length, and exhibiting significant antimicrobial activity against several pathogenic bacterial strains.

## 2. Results

### 2.1. Bioactive Components of Mushroom Extracts

#### 2.1.1. Protein Content

**Figure 1:**
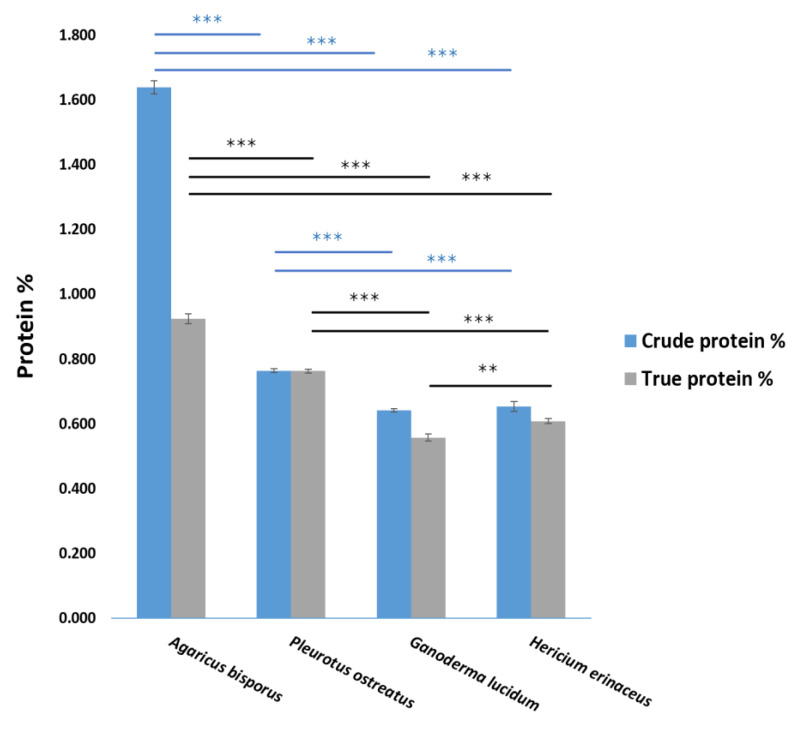
Percentage of crude and true protein content of different mushroom extracts. Data are presented as the mean ± SD. Differences that show statistical significance at ** *p* < 0.01 and *** *p* < 0.001 (n = 3).

To assess protein content, both crude and true protein levels were determined in all mushroom extracts under study. Among the tested species, *Agaricus bisporus* exhibited the highest percentage of crude protein (1.64%) and true protein (0.94%), which was significantly higher (*p* < 0.001) than that of *Pleurotus ostreatus*, which showed 0.76% for both crude and true protein. *Ganoderma lucidum* and *Hericium erinaceus* demonstrated the lowest crude protein content with 0.64% and 0.65%, respectively, with no significant difference observed between them. Similarly, they exhibited the lowest true protein content (0.55% and 0.60%, respectively), with significant differences (*p* < 0.01) between them (Figure 1).

#### 2.1.2. Total Phenols and Total Flavonoids

To assess the bioactive components of the studied mushroom extracts, the total phenol and flavonoid content was determined, expressed as mg gallic acid (GAE) and quercetin (QE) equivalents per gram of sample, respectively (Table 1). Significant variation in both total phenol and flavonoid levels was observed between the four mushroom species. *Pleurotus ostreatus* exhibited the highest phenol content (5.19 ± 0.06 mg GAE/g), followed by *Ganoderma lucidum*, *Agaricus bisporus*, and *Hericium erinaceus* with 2.92 ± 0.04, 2.49 ± 0.08, and 2.01 ± 0.05 mg GAE/g, respectively. For flavonoids, *Agaricus bisporus* demonstrated the highest concentration (1.28 ± 0.02 mg QE/g), followed by *Pleurotus ostreatus*, *Ganoderma lucidum*, and *Hericium erinaceus* with 0.95 ± 0.07, 0.45 ± 0.04, and 0.38 ± 0.02 mg QE/g, respectively.

#### 2.1.3. Phenolic Components

Polyphenols are recognized as the primary antioxidant components within various mushroom species. To characterize these compounds, High-Performance Liquid Chromatography (HPLC) analysis was employed, identifying nine standard phenolic compounds across four different mushroom extracts (Table S1). Notably, all nine phenolic compounds were present in the *Pleurotus ostreatus* extract, including gallic acid (123.62 mg/100 g), catechin (2.88 mg/100 g), chlorogenic acid (6.32 mg/100 g), coumaric acid (2.93 mg/100 g), ferulic acid (3.99 mg/100 g), sinapic acid (16.14 mg/100 g), ellagic acid (5.70 mg/100 g), tannic acid (3.07 mg/100 g), and t-cinnamic acid (2.93 mg/100 g).

However, the presence of these aforementioned compounds varied among the other species. Catechin was notably absent from the *Hericium erinaceus* extract, while coumaric acid was not detected in *Agaricus bisporus*. Furthermore, the *Ganoderma lucidum* extract lacked both ferulic and ellagic acid.

Gallic acid concentrations ranged widely, with the lowest amounts found in *Hericium erinaceus* (0.45 mg/100 g) and *Agaricus bisporus* (1.18 mg/100 g). Conversely, *Ganoderma lucidum* (124.78 mg/100 g) and *Pleurotus ostreatus* (123.62 mg/100 g) exhibited the highest gallic acid levels. Interestingly, *Hericium erinaceus* contained the highest detected amounts of coumaric acid (20.13 mg/100 g), ferulic acid (18.53 mg/100 g), and tannic acid (25.45 mg/100 g) (Table S1).

### 2.2. In Vivo Investigation of Neuroprotective and Hepatoprotective Effects

#### 2.2.1. Evaluation of Body Weights

**Figure 2:**
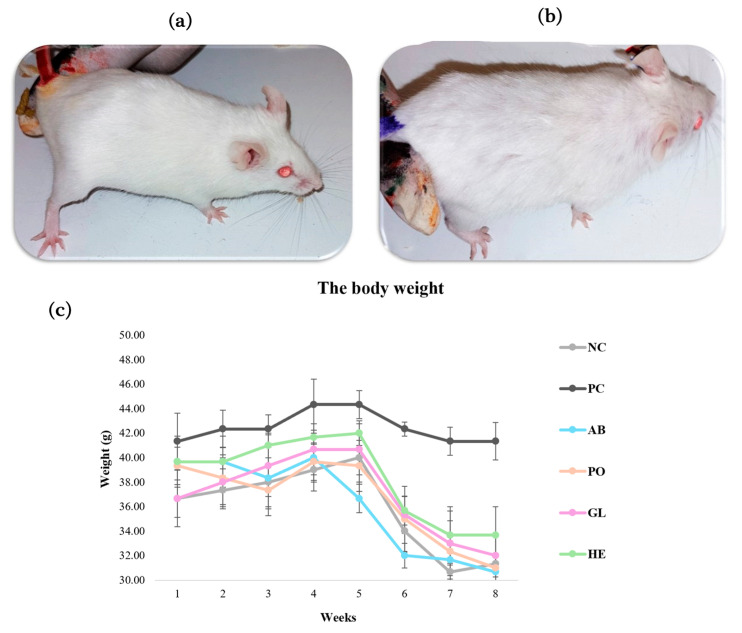
Body weight and physical characterization of aging mice induced by D-gal (120 mg/kg) and treated with different mushroom extracts (500 mg/kg) during the experiment. Where (**a**) the control group observed healthy physical characteristics with smooth, shiny hair, while (**b**) the D-gal group observed rough, curly hair and severe hair loss. (**c**) The changes in body weight of all groups: NC: negative control, PC: positive control, AB: *Agaricus bisporus*, PO: *Pleurotus ostreatus*, GL: *Ganoderma lucidum*, and HE: *Hericium erinaceus* extracts. The D-gal group observed an increase in body weight, while the treated group showed a decrease in body weight. Data are presented as the mean ± SD.

The changes in body weight were measured to assess the impact of D-galactose (D-gal) (120 mg/kg) and mushroom extracts (500 mg/kg) on the mice. Mice were weighed weekly throughout the experiment. The monitored animals displayed differences in their physical appearance at the end of each treatment. Control group mice exhibited smooth, healthy-looking, shiny hair with uniform colors (Figure 2a). In contrast, the PC group (D-gal group) displayed a marked deterioration, with curly, coarse, dull fur, darker patches, and severe hair loss (Figure 2b). Although all groups demonstrated a gradual increase in body weight, no statistically significant differences were observed (Figure 2c). At the end of the eight-week treatment, the control, *Ganoderma lucidum* (GL), *Hericium erinaceus* (HE), *Pleurotus ostreatus* (PO), and *Agaricus bisporus* (AB) groups experienced a dramatic decrease (*p* < 0.05) in body weight, whereas the PC group maintained a stable weight. However, no significant weight differences were detected between the treatments during the experimental period.

#### 2.2.2. Histopathological Studies

**Figure 3:**
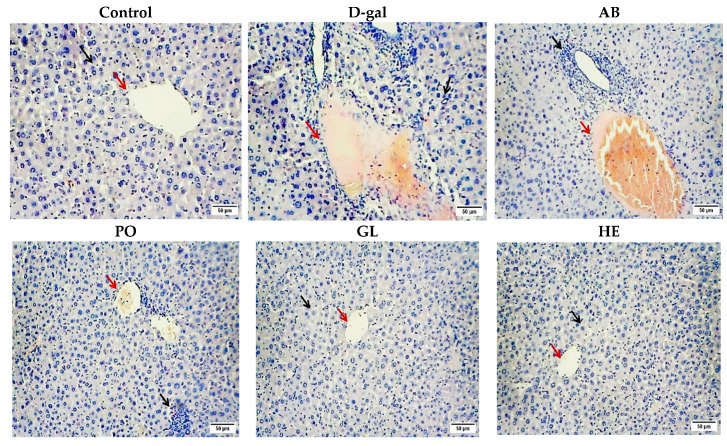
Representative photomicrographs of mouse liver tissues stained with hematoxylin and eosin (H&E) and visualized under a light microscope at 40× magnification. AB: *Agaricus bisporus*, PO: *Pleurotus ostreatus*, GL: *Ganoderma lucidum*, HE: *Hericium erinaceus* extract. The control group exhibited normal hepatic architecture (black arrows), with intact hepatocytes arranged in cords surrounding central veins (red arrows). In contrast, the D-gal group displayed marked hepatic injury characterized by loss of normal architecture, hepatocyte necrosis, congested and dilated portal tracts, hemorrhage (red arrows), and pyknotic nuclei (black arrows). The AB group showed no significant improvement. The PO group demonstrated moderate hepatoprotective effects, with partial restoration of normal hepatic architecture and reduced cellular damage (red arrows). The GL and HE groups exhibited significant hepatoprotective effects, with near-complete restoration of normal hepatic architecture, reduced inflammation, and minimal cellular damage.

**Figure 4:**
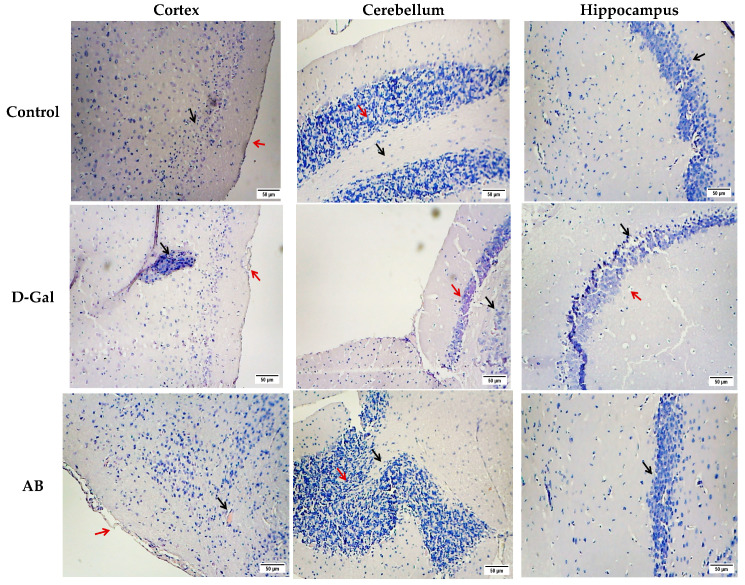

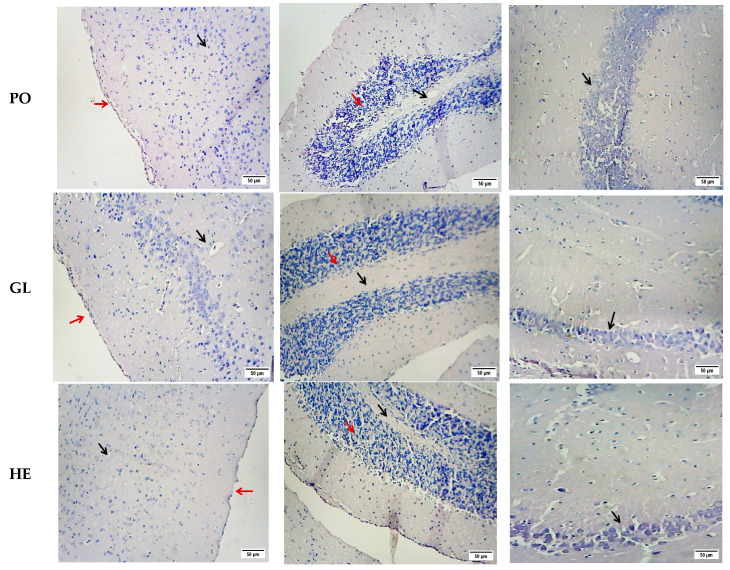
Representative photomicrographs of three brain regions (cerebral cortex, cerebellum, and hippocampus) stained with hematoxylin and eosin (H&E) were examined under a light microscope at 40× magnification. AB: *Agaricus bisporus*, PO: *Pleurotus ostreatus*, GL: *Ganoderma lucidum*, HE: *Hericium erinaceus* extract. The D-gal group exhibited severe neurodegeneration, characterized by loss of normal tissue architecture, necrosis, and vacuolation in all three regions (black arrow), and hemorrhage (red arrows). The AB group showed no significant improvement in the cerebral cortex, but significantly restored the normal structure of the cerebellum and hippocampus. The PO group demonstrated moderate neuroprotection, with near-normal structure in most areas, although some vacuolation and degeneration persisted in cortical cells (black arrow) and showed slight improvement in the cerebellum and hippocampus. The GL group exhibited moderate neuroprotection, with nearly normal structure in the cerebral cortex and restored normal architecture in the cerebellum. However, the hippocampus showed non-significant improvement. The HE group effectively restored the normal structure of all three brain regions.

As depicted in Figure 3 and Figure 4, histopathological examination of liver and brain tissues revealed distinct alterations across the studied groups. The control group displayed normal hepatic architecture (black arrow), characterized by intact hepatocytes and a clear central vein (red arrows), indicating the absence of pathological changes. In contrast, the D-gal group exhibited significant histopathological damage, such as loss of normal architecture, hepatic necrosis, vacuolar degeneration, hemorrhage, and pyknotic nuclei in hepatocytes. While the AB and PO groups showed almost no improvement in liver cell architecture, the GL and HE groups demonstrated more pronounced recovery compared to the D-gal group, which exhibited significant restoration of normal hepatocytic cords and clear central veins, closely resembling the control group (Figure 3). These findings suggest that the GL and HE extracts were more effective in mitigating D-gal-induced liver damage compared to the AB and PO extracts. The histopathological analysis of the liver tissue confirmed the protective effects of the extracts, as evidenced by the quantitative scores (Table 2).

The brain tissue of the negative control mice exhibited normal cerebral cortex, cerebellum, and hippocampus structures. The cerebral cortex displayed a well-defined upper molecular layer, underlying pyramidal cells (black arrow), and a normal pia mater (red arrows) (Figure 4). The cerebellar cortex showed a distinct deep white matter (black arrows) and a granular layer of the cortex (red arrows). The hippocampus demonstrated a regular arrangement of pyramidal cells with minimal cytoplasmic vacuolation and homogeneous tissue appearance (black arrows) (Figure 4). In contrast, the D-gal group displayed severe brain damage across all three regions. The cerebral cortex suffered from extensive vacuolation, degeneration, necrosis, and mononuclear cell infiltration (black arrow), accompanied by meningeal degeneration and hemorrhage (red arrows) (Figure 4). The cerebellum displayed a loss of normal architecture, severe white matter necrosis (black arrows), and a condensed, diminished granular layer (red arrows). The hippocampus was characterized by a complete loss of normal structure, with numerous necrotic and pyknotic neuroglial cells (red arrows) and necrotic pyramidal cells (black arrows) (Figure 4). The AB group showed non-significant improvement in the cerebral cortex but significant improvement in the cerebellum and hippocampus compared to the positive control. The PO group exhibited near-complete recovery in all brain regions. The GL group demonstrated significant improvement in the cerebral cortex and cerebellum, showing a normal cell architecture, while the hippocampus showed a non-significant enhancement of the cell structure. Importantly, HE extract significantly enhanced brain tissue morphology, restoring normal structures in the cerebral cortex, cerebellum, and hippocampus compared to the positive control (Figure 4). The scores in Table 3 provide evidence that the extracts protected brain tissue, which was confirmed by a histopathological analysis.

#### 2.2.3. Biochemical Parameters of Liver Functions

**Figure 5:**
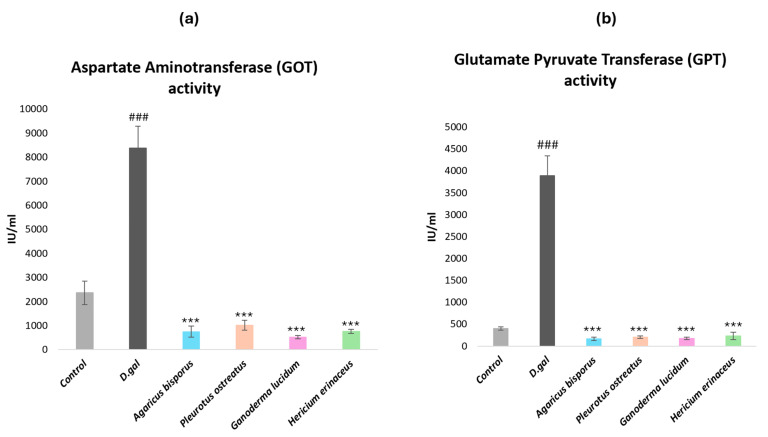
Assessment of serum liver enzymes: (**a**) serum levels of aspartate aminotransferase (AST or GOT) and (**b**) alanine aminotransferase (ALT or GPT) activity in aging mice induced by D-gal (120 mg/kg) and aging mice treated with different mushroom extracts (500 mg/kg). Data are presented as the mean ± SD. Differences that show statistical significance at *** *p* < 0.001 compared with the D-gal group and ^###^ *p* < 0.001 compared with the control group.

To assess hepatic injury in aging mice, serum levels of aspartate aminotransferase (AST or GOT) and alanine aminotransferase (ALT or GPT) were measured. This study investigated the potential hepatoprotective effects of four different mushroom extracts. As expected, aged mice treated with D-galactose (D-gal) exhibited significantly elevated levels of both GOT (Figure 5a) and GPT (Figure 5b) compared to the negative control group (NC) (*p* < 0.001). Administration of the four mushroom extracts (AB, PO, GL, and HE) at a dose of 500 mg/kg significantly reduced both GOT and GPT levels in D-gal-induced aged mice (*p* < 0.001). Notably, while there were no significant differences in GPT levels between the mushroom-treated groups and the control group, there was a significant difference between them in the GOT levels (*p* < 0.05). These findings underscore the detrimental effects of D-gal-induced aging on liver function and highlight the potential hepatoprotective properties of the four studied mushroom extracts.

#### 2.2.4. Molecular Analysis (qRT-PCR)

##### Quantitative Real-Time PCR Analysis of Immunomodulation and Inflammation-Regulation Genes

**Figure 6:**
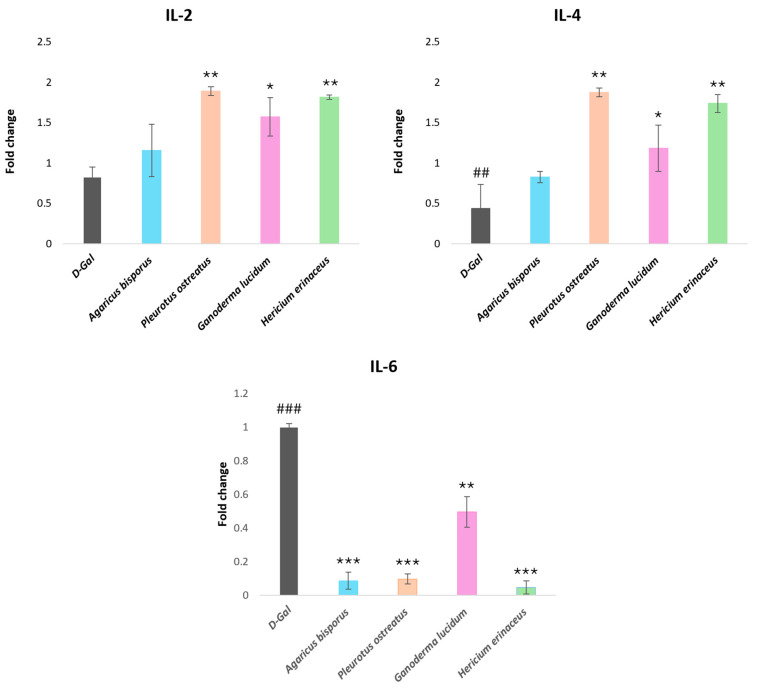
Influence of four mushroom extracts on the levels of cytokine mRNA expression of *IL-2*, *IL-4*, and *IL-6* in the liver of aging mice induced by D-gal (120 mg/kg) and aging mice treated with different mushroom extracts (500 mg/kg). Data are presented as the mean ± SD. Differences that show statistical significance at * *p* < 0.05, ** *p* < 0.01, and *** *p* < 0.001 compared with the D-gal group, ^##^ *p* < 0.01, and ^###^ *p* < 0.001 compared with the control group.

To investigate the impact of aging on immune function and cytokine production, we assessed the gene expression of immunomodulatory cytokines (*IL-2* and *IL-4*) and inflammatory cytokine (*IL-6*) in the liver tissue of mice using qRT-PCR (Figure 6).

Our results revealed a significant downregulation of *IL-2* and *IL-4* gene expression in the D-gal aging model compared to the control group (*p* < 0.01). Conversely, treatment with mushroom extracts significantly upregulated the expression of these cytokines (*p* < 0.05, *p* < 0.01), except for the AB group, which did not show a significant difference. The PO group displayed the highest level of upregulation, followed by the HE group. In contrast, the expression of *IL-6*, a pro-inflammatory cytokine, was significantly elevated in the D-gal aging model (*p* < 0.001) compared with the control group. Treatment with mushroom extracts significantly decreased *IL-6* expression (*p* < 0.001) compared to the aging model, with the HE group demonstrating the most potent inhibitory effect followed by the PO and AB groups displaying no significant differences between them. These findings suggest that mushroom extracts modulate the immune response and reduce inflammation in aging mice (Figure 6).

##### Real-Time PCR Analysis of Neurodegeneration-Related Genes

**Figure 7:**
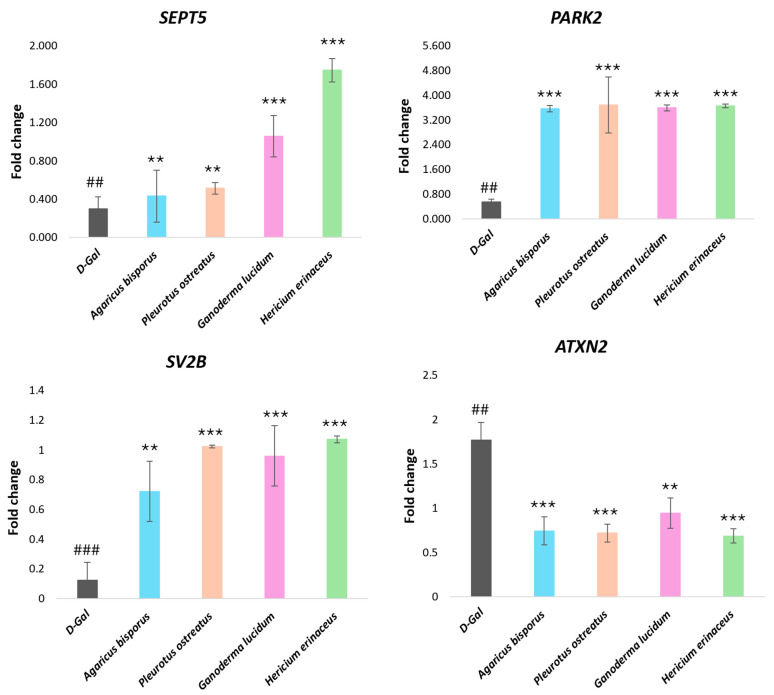
Gene expression differences between four neurodegenerative-related genes in the brain (*SEPT5*, *SV2B*, *ATXN2*, and *PARK2*) of aging mice induced by D-gal (120 mg/kg) and aging mice treated with different mushroom extracts (500 mg/kg). Data are presented as the mean ± SD. Differences that show statistical significance at ** *p* < 0.01, and *** *p* < 0.001 compared with the D-gal group, ^##^ *p* < 0.01, and ^###^ *p* < 0.001 compared with the control group.

To investigate the impact of aging on neurodegenerative processes, we assessed the expression of four genes linked to these disorders (*SEPT5*, *SV2B*, *ATXN2*, and *PARK2*) in a mouse model of aging induced by D-gal. Compared to the control group, the D-gal group exhibited significant downregulation of *SEPT5*, *PARK2*, and *SV2B* (*p* < 0.01) and a significant upregulation of *ATXN2* gene expression (*p* < 0.01), indicative of age-related neurodegeneration (Figure 7).

Subsequently, we evaluated the therapeutic potential of various mushroom extracts in mitigating these age-related changes. Treatment with the mushroom extracts reversed the gene expression patterns observed in the D-gal group. The *SEPT5*, *PARK2*, and *SV2B* gene expression was significantly increased (*p* < 0.001), and the *ATXN2* gene expression was significantly decreased (*p* < 0.001) compared to the D-gal group (Figure 7). The HE extract demonstrated the most potent effect on upregulating SEPT5 gene expression, followed by the GL, PO, and AB groups. Additionally, HE, PO, and GL extracts significantly upregulated *SV2B* gene expression. While no significant differences were observed in *PARK2* and *ATXN2* gene expression among the different mushroom extracts, these findings suggest that mushroom extracts possess therapeutic potential in attenuating age-related neurodegenerative processes.

##### Real-Time PCR Analysis of Genes Encoding Functions Affecting Telomere Length

**Figure 8:**
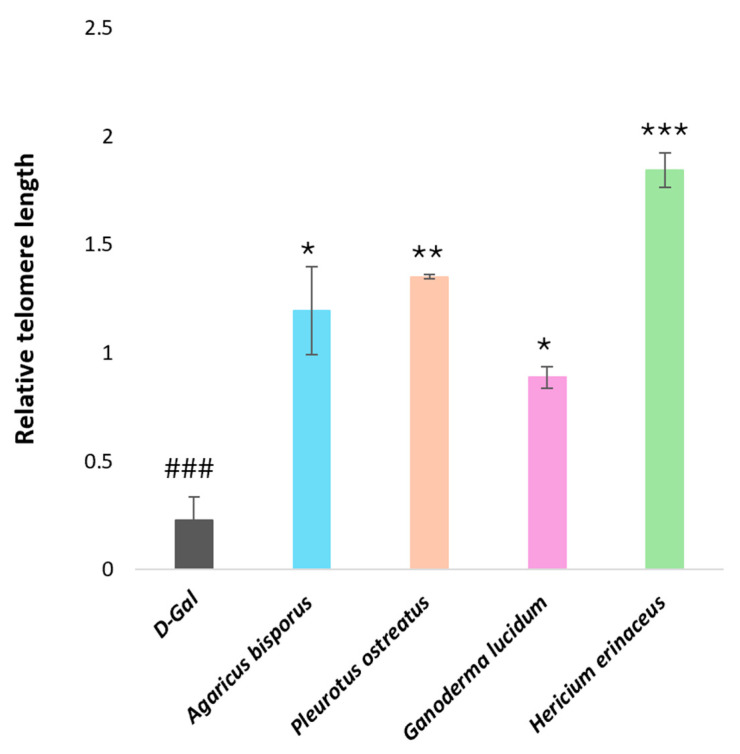
Monitoring of the relative telomere length of aging mice induced by D-gal (120 mg/kg) and aging mice treated with different mushroom extracts (500 mg/kg). Data are presented as the mean ± SD. Differences that show statistical significance at * *p* < 0.05, ** *p* < 0.01, and *** *p* < 0.001 compared with the D-gal group, ^###^ *p* < 0.001 compared with the control group.

We analyzed telomere length gene expression in the mouse brain to investigate the impact of D-galactose-induced aging and the potential anti-aging effects of various mushroom extracts on telomere length. Aged mice (D-gal group) exhibited a significant decrease (*p* < 0.001) in relative brain telomere length compared to the control group. Conversely, all groups treated with mushroom extracts demonstrated a substantial increase in the expression of the genes influencing telomere length compared to the D-gal group. Among the mushroom extracts, the HE extract displayed the most potent protective effect against D-gal-induced telomere shortening (*p* < 0.001), followed by PO, AB, and GL extracts, respectively (Figure 8).

### 2.3. Antimicrobial Activity of Mushroom Extracts

#### 2.3.1. Isolation and Activation of Bacteria

The antimicrobial properties of water extracts from four mushroom species (*Ganoderma lucidum*, *Hericium erinaceus*, *Pleurotus ostreatus*, and *Agaricus bisporus*) were assessed against four pathogenic bacteria, including two Gram-positive bacteria (*Lysinibacillus odyssey* and *Lysinibacillus fusiformis*) and two Gram-negative bacteria (*Klebsiella oxytoca* and *Escherichia coli*) strains. Bacterial cultures were standardized to a turbidity of 0.5 McFarland (1.5 × 10^8^ CFU/mL) before antimicrobial testing (Figure S1).

To comprehensively assess the antibacterial potential of mushrooms, a two-pronged approach was employed. Qualitative antimicrobial activity was determined using the agar well diffusion method, while quantitative assessment was achieved through the minimum inhibitory concentration (MIC) method. This combined approach provided a more complete understanding of the antimicrobial properties of the mushroom extracts.

#### 2.3.2. Antimicrobial Activity Measured by Agar Well Diffusion

The antimicrobial activity of various mushroom extracts was assessed using the agar well diffusion method, with ampicillin (AMP) (10 μg/disk) used as a positive control. The diameter of the inhibition zones (mm) exhibited by 500 mg/mL extracts against the tested bacterial strains is summarized in Table 4 and visualized in Figure S2. The presence of a zone of inhibition (a clear area surrounding the well where bacterial growth is suppressed) signifies antimicrobial activity. A larger zone of inhibition indicates greater antimicrobial activity.

According to the inhibition zone (IZ) size, *Agaricus bisporus* and *Pleurotus ostreatus* exhibited the broadest antimicrobial spectrum, effectively inhibiting all tested bacterial strains. *Agaricus bisporus* was particularly potent against *Lysinibacillus odyssey*, *E. coli*, *Klebsiella oxytoca*, and *Lysinibacillus fusiformis*, with inhibition zones of 22, 20, 15, and 11 mm, respectively. *Pleurotus ostreatus* also demonstrated significant activity, with 21, 18, 14, and 13 mm inhibition zones against the same bacterial strains. *Hericium erinaceus* displayed strong activity against *E. coli* (22 mm) but was less effective against other bacteria. *Ganoderma lucidum* exhibited moderate activity against *E. coli*, *Klebsiella oxytoca*, and *Lysinibacillus odyssey* (16, 15, and 14 mm, respectively) but showed limited activity against *Lysinibacillus fusiformis* (10 mm) (Table 4).

Overall, the results suggest that Gram-negative bacteria were more susceptible to the mushroom extracts than Gram-positive bacteria, likely due to differences in cell wall structure.

#### 2.3.3. Minimum Inhibitory Concentrations (MIC) by Microdilution Method

Furthermore, we employed a standard microdilution assay to determine the minimum inhibitory concentration (MIC) of each extract. This involved a two-fold serial dilution series, ranging from 250 to 1.95 mg/mL. This method provides a precise evaluation of the potency of the mushroom extracts.

According to the lowest concentration of mushroom extracts that prevents visible growth of the microorganism, all extracts displayed antimicrobial activity against *Escherichia coli*, where the MIC value of the *Agaricus bisporus*, *Pleurotus ostreatus*, and *Hericium erinaceus* extracts was 3.90 mg/mL, followed by the *Ganoderma lucidum* extract with a 31.25 mg/mL MIC value. Similarly, *Agaricus bisporus*, *Pleurotus ostreatus*, and *Ganoderma lucidum* inhibited the growth of *Klebsiella oxytoca*, with MIC values of 7.81, 7.81, and 3.90 mg/mL, respectively.

*Agaricus bisporus* proved most effective against *Lysinibacillus odyssey*, requiring the lowest MIC of 1.95 mg/mL. *Pleurotus ostreatus* and *Ganoderma lucidum* followed with MICs of 3.90 and 125 mg/mL, respectively. *Lysinibacillus fusiformis* displayed the highest resistance to all mushroom extracts. *Pleurotus ostreatus* showed the lowest MIC of 15.62 mg/mL, while *Agaricus bisporus* and *Ganoderma lucidum* required 125 mg/mL. *Hericium erinaceus* exhibited no antimicrobial activity against the Gram-positive bacteria *Lysinibacillus fusiformis*, *Lysinibacillus odyssey*, and *Klebsiella oxytoca* (Table 5). The MIC data align with the results obtained from the agar well diffusion assay, providing strong evidence for the antibacterial potential exhibited by the mushroom extracts.

## 3. Discussion

### 3.1. Mushroom Bioactive Components

Oxidative stress, a primary aging driver, accelerates the deterioration of bodily organs and tissues [58]. A simple and effective strategy to mitigate oxidative stress and reduce the risk of age-related diseases is to incorporate natural antioxidant supplements into daily meals [59]. This study investigates the medicinal and bioactive benefits of four prominent edible and medicinal mushrooms: *Ganoderma lucidum* (GL), *Hericium erinaceus* (HE), *Pleurotus ostreatus* (PO), and *Agaricus bisporus* (AB), highlighting their unique contributions to human health and well-being. These species exhibit significant global cultivation and substantial medicinal potential due to their unique bioactive compounds, including polysaccharides, terpenoids, phenols, flavonoids, carotenoids, proteins, minerals, fats, enzymes, and more, which offer a range of therapeutic properties [4]. For example, *Pleurotus* species and *Agaricus bisporus* have antibacterial, antioxidant, anti-cancer, and other therapeutic properties [60,61], while *Ganoderma lucidum* is rich in compounds that combat aging and inflammation [62]. *Hericium erinaceus* contains unique compounds like erinacines and hericenones, which contribute to its neuroprotective and other health benefits [63]. Incorporating these mushrooms or their extracts into daily meals has been shown to help reduce oxidative stress and the risk of age-related diseases [4].

Furthermore, mushrooms exhibit a notable protein content, ranging from 15% to 35% of their dry weight, a value influenced by factors like species, fruiting body morphology, and developmental stage [64]. This protein quality surpasses that of most vegetables, as recognized by the Food and Agriculture Organization (FAO) [65]. The cultivation substrate significantly impacts mushroom protein levels [66].

In our findings, the *Agaricus bisporus* hot water extract demonstrated the highest crude protein (1.64%) and true protein (0.94%) content among the tested species. Conversely, *Ganoderma lucidum* and *Hericium erinaceus* extracts exhibited the lowest levels, ranging from 0.55% to 0.65%. The previous study observed that *A. bisporus* crude protein content on a dry-weight basis has been reported to vary from 19% to 38% [67], and the nitrogen content of different fractions was 19%, 37%, and 11.01% in the Muszynska et al. study [68]. According to Akyuz et al., the protein content of *P. ostreatus* varies depending on the strains and the physical and chemical characteristics of the growth medium [69], where the protein content of mushrooms varies from 12% to 35% dry weight, depending on the species [70]. According to Claude et al. [71], the percentage of *P. ostreatus* protein varied depending on the type of cultivation substrate, ranging from 1.39% to 1.84%. The protein level of dried *G. lucidum* was 7–8%, less than that of many other mushrooms [72]. Liu et al. [14] reported that the crude protein of *G. lucidum* hot water was 23.58%. Research by Friedman [73] found that the dried fruiting bodies of *H. erinaceus* have a dry-weight protein content of about 20.8%.

Bioactive components, particularly the total phenolic and flavonoid content, play a crucial role in the antioxidant properties of mushrooms. Our study analyzed these compounds in hot water extracts from four species of mushrooms, revealing significant variations. Total phenolic and flavonoid contents were expressed as mg gallic acid (GAE/g) and quercetin (QE/g) equivalents per gram of extract. Our results showed that *P. ostreatus* had the highest phenolic content (5.19 ± 0.06 mg GAE/g), while *H. erinaceus* had the lowest (2.01 ± 0.05 mg GAE/g). Conversely, *A. bisporus* had the highest flavonoid content (1.28 ± 0.02 mg QE/g), and *H. erinaceus* had the lowest (0.38 ± 0.02 mg QE/g).

Our findings revealed both similarities and discrepancies with previous research, underscoring the intricate impact of factors such as growth medium, strain, and extraction method. We measured a notably higher phenolic content for *P. ostreatus* (5.19 mg GAE/g) than the value reported by El-Razek et al. (1.78 ± 0.09 mg GAE/g) [39], but our result is consistent with that of Mircea et al. (5.47 ± 0.05 mg GAE/g) [74]. However, these findings contrast with higher values reported elsewhere [75,76]. Similarly, the flavonoid content of our *P. ostreatus* extract (0.95 ± 0.07 mg QE/g) was higher than some reported values (0.10 ± 0.05 mg QE/g [76]; 0.15 mg/g [41]), yet lower than others (2.71 ± 0.06 mg/g [77]; 1.6 mg/g [38]). The results for our *A. bisporus* extract also demonstrated similar variability. Our phenolic content (2.49 ± 0.08 mg GAE/g) was higher than the value reported by Krishnamoorthi et al. (0.441 ± 0.01 mg GAE/g) [78] but lower than values reported in other studies, 5–8 mg GAE/g [79]; 6.43 mg GAE/g [80]; 190.90 ± 0.07 mg GAE/g [81]. Our flavonoid content (1.28 mg QE/g) was also higher than some reported values (1.09 ± 0.02 mg EC/g (catechin equivalents/g) [74] and 0.783 ± 0.03 mg/g [78]) but lower than the value reported by Nitthikan et al. (12.92 ± 0.02 mg QE/g extract) [81]. For *G. lucidum*, our flavonoid content (0.45 ± 0.04 mg QE/g) was close to the value reported by Wu et al. (0.56 mg/g) [82]. Our finding that *G. lucidum* had a higher phenolic content than *H. erinaceus* is consistent with the results reported by Sharpe et al. [83] and Abdullah et al. [84]. The phenolic content of *H. erinaceus* (2.01 mg GAE/g) was lower than the value reported by Abdullah et al. (10.20 ± 2.25 mg GAE/g) [84], but it fell within the range of values reported by Wong et al. (0.26 ± 0.022 to 2.37 ± 0.24 mg GAE/g) [85]. Similarly, our *H. erinaceus* flavonoid content (0.38 mg QE/g) was lower than that reported by Lew et al. (5.31 ± 0.3 mg QE/g) [86]. These discrepancies underscore the importance of considering both extraction methods and growing conditions when interpreting these results. Our study, which used hot water extraction, contributes a specific data point to this body of knowledge.

Our study provides valuable data on the phenolic and flavonoid content of four mushroom species. The comparison between different mushroom extracts highlights the species-specific nature of bioactive compound accumulation. While the results show variations compared to previous studies, these discrepancies emphasize the importance of considering factors such as species, strain, growth conditions, and extraction methodology when evaluating the bioactive potential of mushrooms.

The results of phenolic and flavonoid content corroborate our earlier research, which demonstrated the antioxidant activity of mushroom extracts using DPPH^•^, ABTS^+•^, and FRAP assays. Notably, all extracts, especially at 0.1 mg/mL, exhibited strong antioxidant activity. *P. ostreatus* stood out, displaying the greatest scavenging activity against DPPH and ABTS^+^ radicals (indicated by the lowest IC_50_ values) and the most potent reducing power (lowest EC_50_) among the tested extracts [87].

### 3.2. In Vivo Experimental (Hepatoprotective, Neuroprotection, Immune Modulation)

In the in vivo experimental proceedings, D-galactose induced aging in mice with 120 mg/kg. This concentration of D-gal was selected according to the findings of other researchers’ relevant prior investigations [88]. According to multiple studies, D-gal was shown to have the ability to cause aging-like effects in laboratory animals and cause oxidative damage in the body [46,88]. Thus, D-galactose was used to cause aging in mice, followed by administration, with a 500 mg/kg body weight concentration of the mushroom extract given to the mice. This dose was decided based on the LD_50_ of each type of mushroom extract [89,90,91,92].

One of the often-used metrics for assessing aging models generated by D-gal is body weight. So, in this experiment, the mice’s body weight was recorded weekly. Our findings reveal a significant decrease in body weight (*p* < 0.05) across all mushroom extract groups, while the D-gal group exhibited a sustained increase, potentially indicative of obesity [93,94]. However, studies involving *A. bisporus* polysaccharides have yielded conflicting results, with some showing no significant impact on body weight [46]. Interestingly, the combination of *G. lucidum* and Rhodiola compounds in D-gal-induced aging rats led to a notable increase in body weight in the D-gal group [10].

Aging leads to a decline in brain and liver function, characterized by cellular degeneration and tissue atrophy. Therefore, the histopathological analysis was carried out to investigate the damage in different regions of the mice’s brains, the hepatotoxicity caused by D-gal, and to demonstrate how the various types of mushrooms can improve the therapeutic effect of damage in these organs and tissues. In this study, D-galactose (D-gal) induced significant damage to hepatocytes and three critical brain regions: the cerebral cortex, cerebellar cortex, and hippocampus. These results agree with Li et al. [95], who observed the detrimental effects of D-gal on various tissues, including the accumulation of lipid droplets in the liver, vacuolar dilation, necrosis in the hippocampal region of the brain, and the destruction of skin hair follicles.

However, mushroom-based therapies demonstrated promising protective effects against these age-related impairments, exhibiting varying degrees of hepatoprotective and neuroprotective properties. The AB and PO groups showed minor hepatocyte damage, while the GL and HE groups showed significant liver tissue improvement. All mushroom groups showed encouraging ameliorating benefits against brain tissue damage caused by D-gal. Notably, *Hericium erinaceus* extract exhibited the most significant ameliorative effects. This neuroprotective potential can be attributed to its bioactive compounds, erinacines (A-I) and hericenones (C-H), which readily traverse the blood–brain barrier. These compounds exert potent neurotropic and neuroprotective actions, a key mechanism being the stimulation of nerve growth factor (NGF) synthesis [4]. Previous research has further validated the therapeutic potential of these mushrooms. *P. ostreatus* was able to protect against acute liver injury [34] and restore liver architecture in malnourished mice [96]. *A. bisporus* has demonstrated the ability to reduce D-gal-induced liver damage [95] and even mitigate plaque buildup in Alzheimer’s disease models [97]. *G. lucidum* has exhibited broad-spectrum protective effects against aging-related damage to the brain, liver, and other organs caused by D-gal [10]. It has also shown promise in reducing neurodegenerative disease progression by mitigating oxidative stress, protecting neurons, and enhancing cognitive function [98]. *H. erinaceus* has been highlighted for its ability to protect against age-related cerebellar degeneration [99]. These findings underscore the potential of mushroom-based therapies as a promising approach to combat age-related cognitive decline and liver dysfunction.

Concerning hematological characteristics, the biochemical indicators of liver injury, glutamate pyruvate transaminase (GPT), and glutamate oxaloacetate transaminase (GOT) levels were measured. D-galactose administration significantly elevated GPT and GOT levels in mice, indicative of hepatic damage. Likewise, different studies observed the hepatotoxic effect of D-gal by increasing GPT and GOT levels [95]. Notably, mushroom extracts mitigated this effect (*p* < 0.001), suggesting a hepatoprotective role. These findings are consistent with prior research demonstrating the impact of *P. ostreatus* polysaccharides on obesity, showing a reduction in the levels of GPT and GOT [93]. Furthermore, *A. bisporus* extract treatment has been associated with reduced GPT and GOT activity in murine models [94]. D-gal-induced hepatic injury, particularly evident in aged mice, was significantly ameliorated by *A. bisporus* supplementation [95]. *G. lucidum* ethanol extract observed hepatoprotective effects by reducing the levels of GPT and GOT [100].

On the other hand, aging is associated with a decline in immune function, characterized by increased production of pro-inflammatory cytokines such as *IL-6*; this chronic inflammation has been shown to exacerbate age-related diseases [99,101]. In this study, aging mice exhibited downregulation of the anti-inflammatory cytokines *IL-2* and *IL-4*, while *IL-6* was upregulated. This finding supports the notion that aging compromises the immune system and triggers an inflammatory response within the body. This observation aligns with previous research demonstrating that D-gal-induced aging in animal models results in a heightened inflammatory state, characterized by elevated levels of IL-6 and suppressed levels of IL-2 and IL-4 [3,10]. In contrast, treatment with various mushroom extracts significantly upregulated *IL-2* and *IL-4* expression, suggesting an enhancement of immune function in aged mice. Notably, the PO group displayed the most significant increases in *IL-2* and *IL-4* expression, followed by the HE group. The mushroom extract treatments significantly reduced the *IL-6* expression level, indicating an anti-inflammatory effect. These findings align with previous research demonstrating the beneficial effects of *Hericium erinaceus* on reducing age-related inflammation by reducing the overexpression of *IL-6* [99]. Furthermore, another study confirmed the protective role of *Ganoderma lucidum* against D-galactose-induced aging, characterized by decreased *IL-6* and increased *IL-2* and *IL-4* expression levels [10].

Several recent studies have implicated a range of genes, including *Sv2b*, *Park2*, *Sept5*, and *Atxn2*, in the development of neurological disorders, cognitive impairment, and neurodegenerative diseases such as Alzheimer’s disease (AD) and Parkinson’s disease (PD) [52,53,102]. Among the genes investigated, *SEPT5* showed a significant age-related decline in expression in aging models (*p* < 0.01). This downregulation is particularly concerning given its association with neurodegenerative diseases like Alzheimer’s and Parkinson’s [103,104,105,106,107]. Importantly, treatment with mushroom extracts, particularly *Hericium erinaceus* (HE), significantly reversed this decline by increasing *SEPT5* gene expression. The other extracts (GL, PO, and AB) also demonstrated a potent effect, suggesting that strategies aimed at upregulating *SEPT5* could offer a promising avenue for mitigating age-related neurological impairments. Aging and age-related disorders, such as Parkinson’s disease, are linked to dysfunction of the Parkin protein, which is crucial for breaking down and recycling damaged proteins (proteostasis) and is encoded by the *PARK2* gene [108,109]. Our findings align with this association. We observed a significant downregulation of *PARK2* gene expression in D-gal-induced aged mice (*p* < 0.001). This age-related decline was effectively reversed by treatment with all tested mushroom extracts, with no significant differences in efficacy among the groups. This suggests that the mushrooms’ neuroprotective effects may be mediated, in part, by the restoration of normal *PARK2* expression [110,111]. *SV2B*, a gene crucial for neuronal development, was significantly downregulated in our aged mice (*p* < 0.001). This aligns with previous research linking diminished *SV2B* expression to brain dysfunction associated with aging and other neurotoxins [53,102,112]. Importantly, mushroom extract treatment significantly reversed this effect by upregulating *SV2B* expression (*p* < 0.001), suggesting a neuroprotective role. The *ATXN2* gene, linked to neurodegenerative disorders like spinocerebellar ataxia type 2 (SCA2), is known to exhibit age-dependent increases [113,114,115,116]. Our findings corroborate this, showing elevated *ATXN2* expression in our aging model. Importantly, treatment with mushroom extracts significantly reversed this effect by downregulating *ATXN2* gene expression (*p* < 0.001). This suggests that mushroom extracts may offer a promising avenue for mitigating age-related neurological impairments by targeting genes associated with neurodegeneration. Further research is crucial to elucidate the specific mechanisms involved.

Telomere length, a critical biomarker for biological aging, is known to shorten in various disorders, including those associated with aging and neurodegeneration [117,118,119]. In our study, D-galactose (D-gal) administration significantly decreased relative telomere length in the brains of aged mice (*p* < 0.001), consistent with previous findings on its effect on telomerase activity [120]. Previous investigation into the extracts of *A. bisporus*, *P. ostreatus*, *H. erinaceus*, and *G. lucidum* revealed a significant protective effect against D-gal-induced telomere shortening. This protection was attributed to the observed upregulation of telomere length genes [87]. Treatment with all mushroom extracts significantly reversed this D-gal-induced telomere shortening (*p* < 0.001). *Hericium erinaceus* (HE) demonstrated the most pronounced protective effect, followed by the PO, AB, and GL groups. These results align with a recent study, which showed that triterpenoids from *Ganoderma lucidum* were able to increase telomere length in mouse brains [121]. Protective effects are likely due to the mushrooms’ rich content of bioactive compounds, such as antioxidants, anti-inflammatory agents, and polysaccharides. These compounds were suggested to mitigate oxidative stress and stimulate telomerase activity, thereby preventing telomere shortening [122,123,124]. These findings indicate that mushroom consumption offers a promising dietary approach for mitigating age-related telomere shortening and associated health declines. However, further research is needed to fully understand the molecular pathways involved.

### 3.3. In Vitro Experimental (Antimicrobial Activity)

To comprehensively assess the antimicrobial potential of the aqueous mushroom extract, two standard methods were employed: the agar well diffusion method and the determination of minimum inhibitory concentrations (MICs). The agar well diffusion assay provided preliminary insights into the antimicrobial activity by evaluating the zone of inhibition around wells containing the extract. Subsequently, MIC determinations were conducted to quantify the minimum extract concentration required to inhibit microbial growth. Comparative analysis of the results obtained from both methods served to corroborate and refine the observed antimicrobial activity of the mushroom extracts. Our results revealed that all mushroom extracts exhibited antimicrobial activity against four pathogenic bacteria (*Escherichia coli*, *Klebsiella oxytoca*, *Lysinibacillus odyssey*, and *Lysinibacillus fusiformis*), except for *H. erinaceus* extract, which was only effective against *E. coli*, aligning with previous reports, where the water extract of *H. erinaceus* did not show antibacterial activity against Gram-positive bacteria [27].

*H. erinaceus* extract showed the maximum inhibition zone (22 mm) against *E. coli*, while the minimum was seen with *G. lucidum* against *Lysinibacillus fusiformis* (10 mm). The lowest minimum inhibitory concentration (MIC) value was recorded in *A. bisporus* extract against *Lysinibacillus odyssey* (1.95 mg/mL), and the highest MICs were observed in *A. bisporus* and *G. lucidum* extracts against *L. fusiformis* and *L. odyssey* (125 mg/mL). Overall, *A. bisporus* and *P. ostreatus* demonstrated the most potent antibacterial activity, while *L. fusiformis* was the most resistant bacterium.

These findings align with prior studies that show polar solvents like water, acetone, and ethanol are more effective for extracting antimicrobial compounds from mushrooms compared to non-polar solvents [48]. For instance, previous reports confirmed that aqueous extracts of *A. bisporus* and *P. ostreatus* were highly effective against bacteria like *E. coli* and other strains [48,125]. *P. ostreatus* aqueous and ethanolic extracts, specifically, have been shown to have inhibition zones ranging from 18.5 to 23.25 mm against *E. coli*, with low MIC values (7.81–15.62 mg/mL) [39,126]. Similarly, *G. lucidum* and *H. erinaceus* extracts have demonstrated broad-spectrum antibacterial activity against various pathogens [18,27,28,29,127]. The antibacterial effects of these mushrooms have been attributed to their rich content of bioactive compounds, including tannins, flavonoids, phenols, and triterpenoids [17,18,26,42,48]. The results suggest that edible and medicinal mushrooms have strong potential as natural antibacterial agents and functional foods. However, further research is essential to isolate the specific compounds responsible for this activity and to determine optimal dosages and administration routes for their effective use.

## 4. Materials and Methods

### 4.1. Collection of Samples

Four types of mushrooms were used in this study: fresh edible *Agaricus bisporus* (AB) and *Pleurotus ostreatus* (PO), sourced from the Agriculture Research Centre, Alexandria, Egypt (ARC), in October 2021, and processed immediately after harvest. Additionally, dried medicinal *Ganoderma lucidum* (GL) and *Hericium erinaceus* (HE) were procured from DXN Company, Petaling Jaya, Malaysia.

### 4.2. Preparation of Aqueous Extract

The fresh mushrooms were sliced thinly, lyophilized (−80 °C) (Labocon, Hampshire, UK), and then processed into fine powder; 100 mL of hot, sterile distilled water (80 °C) was mixed with 4 g of powdered mushroom sample for 3 h while stirring (Gallenkamp, Cambridge, UK). The mixture was then incubated overnight at 25 °C (Heraeus, Hanau, Germany) [128]. The suspension was centrifuged at 2147× *g* for 15 min at RT (Gemmy, Taiwan, China), and then the supernatant was filtered with filter paper. Small aliquots of the extracts were stored at −20 °C (Zanussi, Pordenone, Italy). For the antibacterial experiments, the extract was concentrated by lyophilization and dissolved in 1% DMSO (*v*/*v*) according to the appropriate amount used.

### 4.3. Chemical Analysis of Mushroom Extracts

#### 4.3.1. Determination of Protein Content

The true protein (the actual quantity of protein) and crude protein (all nitrogen sources, including non-protein nitrogen) levels in the hot water mushroom extracts were measured using the Kjeldahl method [129] (RAYPA Kjeldahl distiller, Barcelona, Spain).

#### 4.3.2. Determination of Total Flavonoid Content

The total flavonoid content in the aqueous mushroom extracts was determined using the aluminum chloride colorimetric assay, as described by Zou et al. [130]. The absorbance of each sample was measured spectrophotometrically (PG Instruments Limited, Leicestershire, UK) at 415 nm. A standard curve of quercetin (QR) (0.1–1.0 mg/mL) was used to calculate the flavonoid concentration in each extract, with results expressed as milligrams of quercetin equivalent per 100 g of the sample.

#### 4.3.3. Determination of Total Phenolic Content

The Folin–Ciocalteu method, as described by Singleton et al. [131], was employed to determine the total phenolic content in aqueous mushroom extracts. The absorbance of each sample was measured spectrophotometrically at 765 nm. This involved the use of a gallic acid standard curve, ranging from 0.01 to 1.0 mg/mL, to quantify the concentration of total phenolic compounds. The results were subsequently expressed as mg of gallic acid equivalents (GAEs) per gram.

#### 4.3.4. Determination of Individual Phenolic Components

To identify and quantify the phenolic compounds in mushroom extracts, a high-performance liquid chromatograph (HPLC) Agilent 1260 Infinity system (Agilent Technologies, Waldbronn, Germany) was used. The system was equipped with an Agilent Multiple Wavelength Detector (MWD) and an autosampler with a 100 μL sample loop. This method is described by Roberts et al. (2018) [132].

Chromatographic separation was performed on a ZORBAX Eclipse Plus C18 analytical column (Agilent Technologies, Santa Clara, CA, USA) (100 × 4.6 mm, 3.5 µm particle size); 5 μL of the mushroom extract was injected into the system. The column temperature was maintained at a constant temperature (30 °C), and the flow rate was set to a specific value (1.0 mL/min). The mobile phase consisted of a gradient elution of two solvents (Solvent A: water with 2% acetic acid; Solvent B: acetonitrile).

Polyphenolic compounds were detected at a wavelength of 280 nm. Nine commercial phenolic compounds were used as standards (gallic acid, catechin, chlorogenic acid, coumaric acid, ferulic acid, sinapic acid, ellagic acid, tannic acid, and t-cinnamic acid). Data were managed using HP ChemStation software, version G1701AA.

### 4.4. Neuroprotective and Hepatoprotective Effects: An In Vivo Study

#### 4.4.1. Animal Housing and Experimental Design

**Figure 9:**
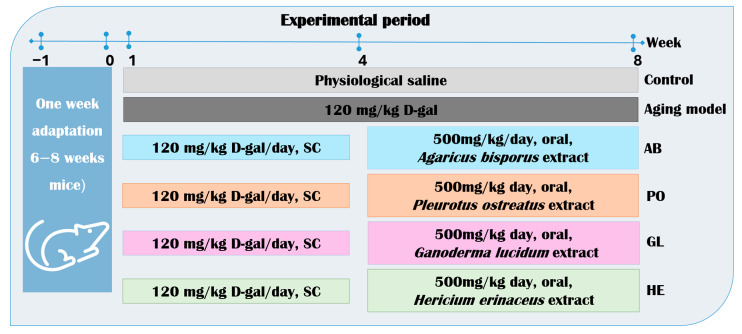
Illustration providing an overview of the animal experimental timeline. Aging was induced with 120 mg/kg D-galactose subcutaneously (SC) for four weeks, followed by treatment with 500 mg/kg of various mushroom extracts orally for four weeks.

Ninety male mice (6–8 weeks, 30–45 g) were randomly and evenly divided into 6 groups; each group had 5 mice in a cage, and each group had 3 replicates in individually ventilated cages (Optimice IVC, Animal Care Systems, Centennial, CO, USA), under controlled conditions: 12 h light/dark cycle, 20–26 °C, and 40–60% humidity. Animals receive a standard diet and water. All procedures were approved by the Animal Experiment Ethics Committee of Alexandria University (ALEXU-IACUC) (approval number: AU082301243124). Animal housing and euthanasia were performed according to the recommendations of the Guide to the Care and Use of Laboratory Animals. The experimental groups were divided as follows: the negative control (NC) received once-daily subcutaneous (SC) injections of normal saline, and the positive control (PC) (aging mice) received daily SC injections of D-galactose (120 mg/kg) for 8 weeks to induce aging [133]. Four groups were treated with different mushroom extracts: *Agaricus bisporus* mushroom extract group (AB), *Pleurotus ostreatus* mushroom extract group (PO), *Ganoderma lucidum* mushroom extract group (GL), and *Hericium erinaceus* mushroom extract group (HE), which received daily SC injections of D-gal (120 mg/kg) for 4 weeks (from week 1 to 4) followed by oral administration of the mushroom extract (500 mg/kg) for 4 weeks (from week 5 to 8) (Figure 9). Mushroom extract dosages were determined based on previously established LD_50_ values [89,90,91,92]. The change in body weight of mice was recorded weekly throughout the experiment.

#### 4.4.2. Blood and Tissue Collection

After the end of the 8-week experiment, all mice fasted for 12 h and were sacrificed via ether inhalation. Blood was collected by retro-orbital bleeding into a plain tube and centrifuged at 2000× *g* for 10 min to separate and remove serum. The collected serum was subjected to liver function parameters such as aspartate aminotransferase (GOT) and glutamic pyruvic transaminase (GPT) using the specific kits (Giesse Diagnostics, Guidonia Montecelio, Italy), following the manual protocol, and was performed in triplicate (Microlab 300, DIrene, The Netherlands). The liver and brain were excised from the animal, weighed immediately, and rinsed in saline. Part of these tissues was transferred to RNA-later and stored at −20 °C for RNA extraction, and the other part was immersed in 10% neutral buffered formalin and stored for histopathological examination. All procedures were approved by the Faculty of Medicine of the University of Alexandria, Egypt.

#### 4.4.3. Pathological Examination of Tissues

Liver and brain tissues were embedded in paraffin. Tissue sections (3–5 microns thick) were cut and stained with hematoxylin and eosin (H&E) according to Bancroft and Gamble [134]. All observations were subjected to light microscopy for histopathologic evaluation at 40× magnification. A semi-quantitative scoring system was used to objectively evaluate the extent of tissue damage. All analyses were performed by an observer who was blinded to the experimental groups.

#### 4.4.4. RNA Isolation and Quantitative PCR

Total RNA was extracted from liver and brain tissues utilizing Trizol according to the instructions provided in the manual (GENEzol Reagent, Geneaid, New Taipei, Taiwan), and complementary DNA (cDNA) was produced using reverse transcriptase (Enzynomics, Daejeon, Republic of Korea). The cDNA products were employed directly with the SYBR Green mixture (Enzynomics, Daejeon, Republic of Korea) [135]. To analyze gene expression, specific primer sets were chosen to investigate the molecular mechanisms underlying D-galactose-induced damage and the potential neuroprotective and anti-aging effects of mushroom extracts. The selected genes were categorized into three functional groups: Nerve-related genes, such as septin-5 (*SEPT5*), synaptic vesicle glycoprotein 2B (*SV2B*), ataxin-2 (*ATXN2*), and parkin RBR E3 ubiquitin protein ligase (*PARK2*), were chosen to evaluate neuronal health and function. These genes are involved in synaptic regulation, cerebellar ataxias, and Parkinson’s disease, all of which are relevant to the neurological deficits associated with D-galactose-induced aging. Cytokine genes: We analyzed the expression of cytokine genes, including interleukin-2 (*IL-2*), interleukin-4 (*IL-4*), and interleukin-6 (*IL-6*). These cytokines play a crucial role in mediating the inflammatory response, which contributes to the neurodegeneration and systemic aging triggered by D-galactose. Telomere length genes: We selected the telomere and 36B4 genes to evaluate the effects of the extracts on cellular aging. Telomere length is a key biomarker of aging, and preserving it is a primary mechanism by which certain compounds can exert anti-aging effects. The 36B4 gene was used as a reference gene for telomere length measurement. The primer sequences for these genes were obtained from references [107,119,136], and are presented in Table S2. The PCR process was conducted at 95 °C for 10 to 15 min, followed by 30 to 45 cycles at 94 °C for 10 to 15 s, 60 °C for 30 to 60 s, and 72 °C for 15 to 60 s. The expression levels were assessed using the 2^−ΔΔCt^ method [137], with GAPDH serving as the endogenous reference gene [135]. Telomere lengths were evaluated by calculating the telomere/single-copy gene ratio (T/S ratio). The relative telomere ratio (T/S) is represented as 2^−ΔΔCt^, where ΔCt is defined as Ct (telomere) − Ct (36B4) [138].

### 4.5. Determination of Antimicrobial Activity

#### 4.5.1. Preparation of Bacterial Isolates and Inoculum

Four pathogenic bacterial strains, previously isolated from infected rabbit stomachs at the Department of Genetics, Faculty of Agriculture, Alexandria University, were identified using conventional methods, including colony morphology and Gram staining. These strains included two Gram-positive bacteria: *Lysinibacillus odyssey* (NR025258.1) and *Lysinibacillus fusiformis* (LT223594.1), and two Gram-negative bacteria: *Klebsiella oxytoca* (KP893565.1) and *Escherichia coli* (KP7893332.1). Bacterial identification was confirmed by sequencing the 16S rRNA (ribosomal RNA region) [139]. To prepare inocula, individual colonies of each strain were streaked onto LB (Luria-Bertani) agar plates and incubated at 37 °C overnight. A single colony was then transferred to 5 mL of LB broth and incubated at 37 °C overnight to obtain pure cultures. The turbidity of each bacterial suspension was adjusted to a standard MacFarland 0.5 (1.5 × 10^8^ CFU/mL) using a spectrophotometer (PG Instruments Limited, Leicestershire, UK) at a wavelength of 660 nm.

#### 4.5.2. Agar Well Diffusion Method

To qualitatively assess the antimicrobial activity of mushroom extracts, the agar well diffusion method was employed [140]. Ampicillin (AMP) at 10 µg/disk is used as a standard antibiotic. The bacterial suspensions were prepared at a concentration of 1.5 × 10^8^ CFU/mL and inoculated onto the center of solidified Violet Red Bile Lactose (VRBL) agar plates. Wells with a diameter of 8 mm were punched into the agar by a sterile cork borer, and 100 µL from each type of extract (500 mg/mL) was poured into each well of all 4 different bacteria, and the AMP 10 µg disc was added. The plates were incubated at 4 °C for one hour to allow diffusion of the extract into agar, then the plates were incubated overnight at 37 ± 2 °C. This experiment was conducted in triplicate. After 24–48 h, the diameters of the inhibition zones, including the well diameter, were measured in millimeters (mm). The average values and standard deviation (SD) were calculated for the three replicates.

#### 4.5.3. Microdilution Method

The minimum inhibitory concentration (MIC) of mushroom extracts was determined using a standardized microdilution assay in 96-well microplates. This method quantitatively assesses the lowest concentration of mushroom extracts that effectively inhibits the growth of the target bacteria [79]. A two-fold serial dilution of each extract, ranging from 250 to 1.95 mg/mL, was prepared in the microplate wells. A bacterial suspension of 1.5 × 10^8^ CFU/mL was added to each well, along with positive and negative controls. Positive controls consisted of bacterial suspension and media, while negative controls contained only media. Blank wells filled with blank plate solution (BPS) were included for background correction during plate reading.

The microplates were incubated at 37 °C for 18–24 h and then read using an ELISA reader (Robonik Readwell Touch, Ambernath, India). The MIC was defined as the lowest concentration of the extract that inhibited visible bacterial growth. The average MIC values and standard deviations (SDs) were calculated from three independent replicates.

### 4.6. Statistical Analysis

Data are presented as mean ± standard deviation (SD). Statistical analysis was performed using one-way and two-way analysis of variance (ANOVA) tests, followed by Tukey’s HSD post hoc test for multiple comparisons, using SPSS software version 25 (SPSS Inc., Chicago, IL, USA). Differences were considered statistically significant at *p* < 0.05 and *p* < 0.01. Gene expression data were analyzed using the 2^−ΔΔCt^ method [135].

## 5. Conclusions

In conclusion, the studied mushrooms (*Ganoderma lucidum*, *Hericium erinaceus*, *Pleurotus ostreatus*, and *Agaricus bisporus*) exhibit a promising array of health benefits, including anti-aging, anti-inflammatory, and antibacterial properties. Our phytochemical analysis suggests that mushroom extracts may offer protection against oxidative damage. Their potential as natural antioxidants and neuroprotective/hepatoprotective agents has positioned them as valuable candidates for the “nutra-mycoceuticals” segment. By modulating key genes involved in immune response, inflammation, neuroprotection, and cellular aging, these fungi offer a bio-based approach to address pressing health concerns and are able to contribute to bio-based research and One Health applications for societal advancement. Notably, all studied mushrooms demonstrated significant antimicrobial activity against *E. coli*, and *Lysinibacillus fusiformis* was demonstrated to be the most resistant bacterium to all mushroom extracts. *Agaricus bisporus* and *Pleurotus ostreatus* extracts were particularly effective against various bacterial strains, highlighting their potential as functional foods with antibacterial properties. Beyond their direct health benefits, these mushrooms offer a sustainable solution to environmental and agricultural challenges. By repurposing agricultural waste and reducing reliance on synthetic chemicals, they contribute to an eco-friendly future, emphasizing the deep interconnectedness of human health, ecological sustainability, and modern agricultural practices. However, further research is imperative to elucidate their protective mechanisms and optimize their therapeutic potential fully. Standardized protocols for disease management and drug development are essential to harness the full power of these medicinal fungi.

## Figures and Tables

**Table 1:** Total phenols and total flavonoids of mushroom extracts.

Mushroom Sample	*Agaricus bisporus*	*Pleurotus ostreatus*	*Ganoderma lucidum*	*Hericium erinaceus*
Total Phenols (mg GAE/g Extract)	2.49 ± 0.08	5.19 ± 0.06	2.92 ± 0.04	2.01 ± 0.05
Total Flavonoid (mg QE/g Extract)	1.28 ± 0.02	0.95 ± 0.07	0.45 ± 0.04	0.38 ± 0.02

GAE: gallic acid equivalent. QE: quercetin equivalent. Data are presented as the mean ± SD (n = 3).

**Table 2:** Histological scores in the liver.

Groups	Sinusoidal Dilatation	Vacuolar and Hydropic Degeneration	Hemorrhage	Mononuclear Cell Infiltration (Inflammation)
Control	−	−	−	−
D-gal	−	++	+++	++
AB	−	−	++	++
PO	−	−	+	+
GL	−	−	−	−
HE	−		−	−

− No histopathologic change. + Histopathology in <20% of fields. ++ Histopathology in 20 to 60% of fields. +++ Histopathology in >60% of fields.

**Table 3:** Histological scores in the brain.

Groups	Cerebral Cortex					Cerebellum		
	Vaculation	Necrosis	Corrugation of the Meninges	Hemorrhage	Inflammation	Vaculation	Necrosis	Hemorrhage
Control	−	−	−	−	−	−	−	−
D-gal	+++	+++	+++	+++	+++	+++	+++	−
AB	++	++	++	+	++	−	−	−
PO	+	+	+	−	+	++	++	−
GL	+	+	−	−	+	−	−	−
HE	−	−	−	−	+	−	−	−

− No histopathologic change. + Histopathology in <20% of fields. ++ Histopathology in 20 to 60% of fields. +++ Histopathology in >60% of fields.

**Table 4:** The inhibition zones (mm) of different types of mushroom extracts against four pathogenic bacterial strains were determined by using the agar well diffusion method.

Diameter of Inhibition Zone (mm)					
Microorganism	Mushroom Extract (500 mg/mL)				Ampicillin (AMP) (10 μg/disk)
	Agaricus bisporus	Pleurotus ostreatus	Ganoderma lucidum	Hericium erinaceus	
**Gram-negative bacteria**					
*Escherichia coli*	20.00 ± 0.15	18.00 ± 0.06	16.00 ± 0.10	22.00 ± 0.06	24
*Klebsiella oxytoca*	15.00 ± 0.10	14.00 ± 0.10	15.00 ± 0.10	-	24
**Gram-positive bacteria**					
*Lysinibacillus odyssey*	22.00 ± 0.06	21.00 ± 0.06	14.00 ± 0.06	-	23
*Lysinibacillus fusiformis*	11.00 ± 0.06	13.00 ± 0.06	10.00 ± 0.06	-	25

(-) = no inhibition zone. Data are presented as the mean ± SD (n = 3).

**Table 5:** Minimum inhibitory concentration (MIC) of mushroom extracts against pathogenic Gram-negative and Gram-positive bacteria.

Microorganism	MIC Value (mg/mL)			
	*Agaricus bisporus*	*Pleurotus ostreatus*	*Ganoderma lucidum*	*Hericium erinaceus*
**Gram-negative bacteria**				
*Escherichia coli*	3.90 ± 0.33	3.90 ± 0.52	31.25 ± 0.23	3.90 ± 0.4
*Klebsiella oxytoca*	7.81 ± 0.38	7.81 ± 0.46	3.90 ± 0.16	-
**Gram-positive bacteria**				
*Lysinibacillus odyssey*	1.95 ± 0.24	3.90 ± 0.27	125.00 ± 0.19	-
*Lysinibacillus fusiformis*	125.00 ± 0.57	15.62 ± 0.55	125.00 ± 0.28	-

(-) = no results. Data are presented as the mean ± SD (n = 3).

## Data Availability

The original contributions presented in this study are included in the article and Supplementary Materials. Further inquiries can be directed to the corresponding authors.

## Supplementary Materials

The following supporting information can be downloaded at: https://www.mdpi.com/article/10.3390/ijms26178440/s1. References [141,142,143,144,145,146,147,148,149,150,151,152,153,154,155,156,157,158] are cited in Supplementary Materials.
