# Supplementary material for: Investigating the Neuroprotective, Hepatoprotective, and Antimicrobial Effects of Mushroom Extracts
Source: Int J Mol Sci. 2025 Aug 29;26(17):8440. doi: 10.3390/ijms26178440 (PMC12429343; doi:10.3390/ijms26178440)
Supplement: Supplementary file 1 [file ijms-26-08440-s001.zip › ijms-3825233-supplementary.pdf]

Table S1. Phenolic acid composition of mushroom extracts.

Compound	Concentration of phenolics (mg/100g)				Activity	References
	Agaricus bisporus	*Pleurotus ostreatus*	*Ganoderma lucidum*	*Hericium erinaceus*		
**Gallic acid**	1.18	123.62	124.78	0.45	Anti-Oxidation, Neuroprotective, Anti-Inflammation, and Antimicrobial	[141–143]
**Catechin**	5.79	2.88	4.78	ND	Anti-inflammatory, Anti-neuroprotective, Antimicrobial, and Hepatoprotective	[144]
**Chlorogenic**	3.90	6.32	3.75	4.45	Anti-Inflammation, Anti-Oxidation, Hepatoprotection, Neuroprotection, and Antimicrobial Effects	[145]
**Coumaric**	0.00	2.93	3.11	20.13	Anti-Oxidation and Antimicrobial	[146,147]
**Ferulic**	9.23	3.99	0.00	18.53	Anti-Inflammation, Anti-Oxidation, Anti-Apoptosis, and Anti-fibrosis	[148,149]
**Sinapic**	3.97	16.14	4.94	9.46	Anti-Inflammation, Anti-oxidation, Neuroprotective, Hepatoprotective, and Antibacterial	[150,151]
**Ellagic**	13.91	5.70	0.00	9.77	Antimicrobial, Anti-aging, Anti-Inflammatory, and Hepatoprotective	[152–154]
**Tannic**	11.81	3.07	10.38	25.45	Anti-Inflammation, Anti-Oxidation, and Antimicrobial	[155,156]
**T-cinnamic**	2.28	2.93	4.96	4.68	Anti-Inflammation, Antimicrobial, Neuroprotective, and Anti-Inflammation	[157,158]

ND, no date (n=3).

Table S2. RT-PCR primer design sequence.

Primers	Forward primer	Reverse primer	Accession no.	References
SEPT5	GAC CCC AGA GGA CAA ACA GG	ACC ATG AGC GTG AAG TCG AA	NM_213614	[107]
SV2B	TGC TGG AGA TGG GCA AAC AT	TGA ACA CCT TTT CCG GGG TC	NM_153579	
ATXN2	CCC GGG CGT ACA ACC TTT AT	TGT CGC TGT TGG GGC ATA TT	NM_009125	
PARK2	ACC CAC CTA CAA CAG CTT TTT C	CAG CAA GAT GGG CCC TGG	NM_016694	
IL-2	GGA AAC ACA GGA ACA ACT GGA	TTC AAT TCT GTG ACC TTC TTG G	AF068057	[136]
IL-4	AGA GCT CGG TGA CCT CAG AC	CTT GCA TGG CGG TCT TTA G	DQ852343	
IL-6	GAA AAC ACC AGG GTC AGC AT	CAG CCA CTG GTT TTT CTG CT	AF169176	
GAPDH	ATC AAG TGG GGT GAT GCT GGT	CCT GCT TCA CCA CCT TCT TGA	L23961	[135]
Telomere	CGG TTT GTT TGG GTT TGG GTT TGG GTT TGG GTT TGG GTT	GGC TTG CCT TAC CCT TAC CCT TAC CCT TAC CCT TAC CCT	[119]	
36B4-R	ACT GGT CTA GGA CCC GAG AAG	TCA ATG GTG CCT CTG GA G ATT		

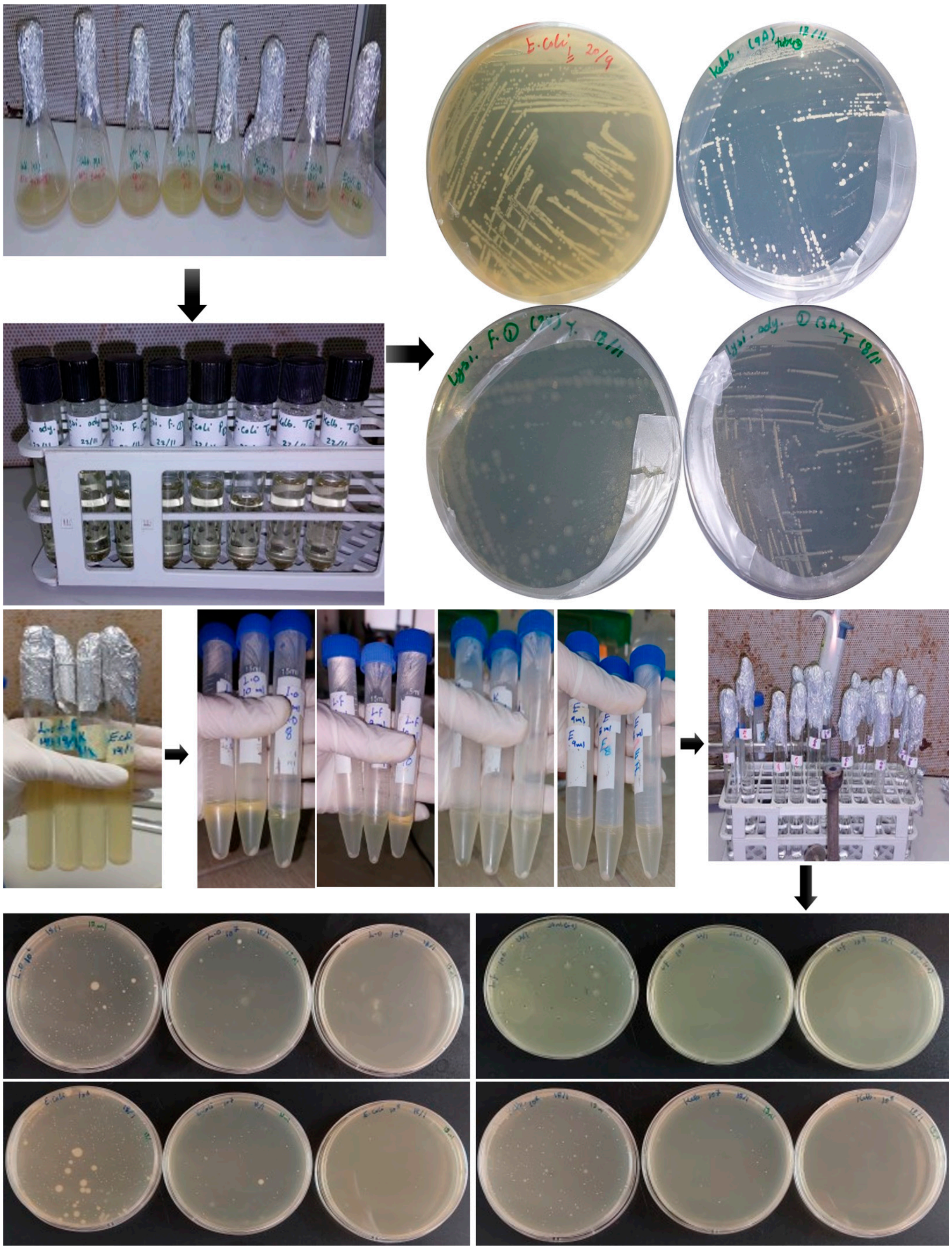

Figure S1. Illustrating the results of the activation, purification, and dilution processes of the bacterial strains under study. Where E. coli (*Escherichia coli*), Kleb. (*Klebsiella oxytoca*), L.f (*Lysinibacillus fusiformis*), and L.o (*Lysinibacillus odyssey*). The bacteria were isolated and incubated overnight at 37 °C, then diluted up to the 8th dilution to reach the parametric standard of MacFarland 0.5 (1.5 × 10^8 CFU/mL). They were then distributed in petri dishes and incubated overnight at 37 °C to determine the appropriate dilution for each strain.

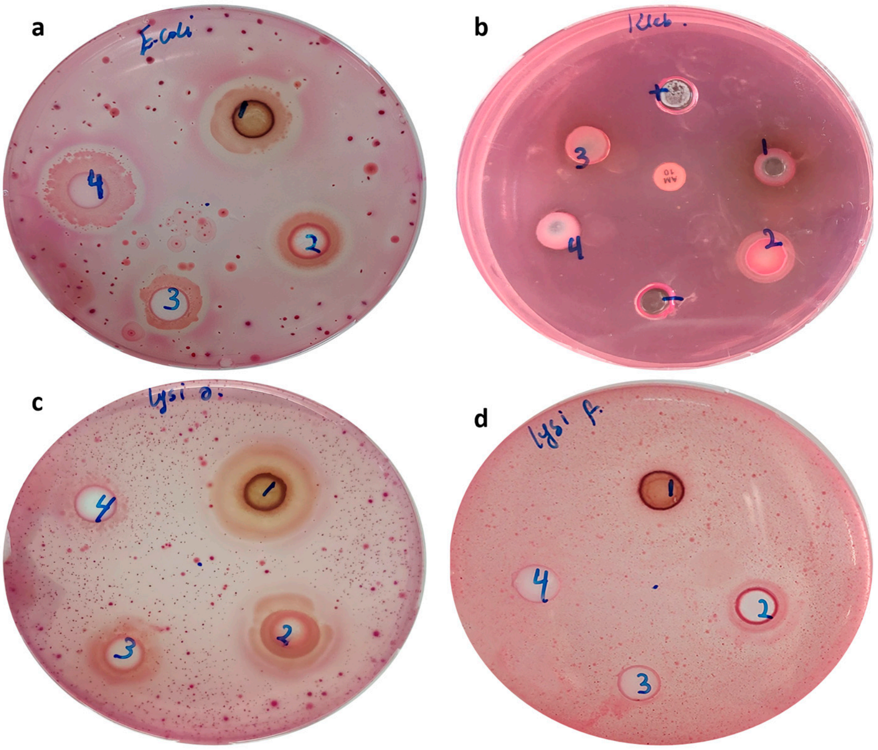

Figure S2. Petri plates showing the antimicrobial activity of different types of mushroom extracts (500 mg/mL) using the Agar well diffusion method against 4 different pathogenic bacterial strains. Where, a: (*Escherichia coli*), b: (*Klebsiella oxytoca*), c: (*Lysinibacillus odyssey*), and d: (*Lysinibacillus fusiformis*). Also: 1: AB (*Agaricus bisporus*), 2: PO (*Pleurotus ostreatus*), 3: GL (*Ganoderma lucidum*), and 4: HE (*Hericium erinaceus*). The diameter of the inhibition zone was measured in mm.
